# DNA Replication Licensing Protein MCM10 Promotes Tumor Progression and Is a Novel Prognostic Biomarker and Potential Therapeutic Target in Breast Cancer

**DOI:** 10.3390/cancers10090282

**Published:** 2018-08-22

**Authors:** Ravikiran Mahadevappa, Henrique Neves, Shun Ming Yuen, Muhammad Jameel, Yuchen Bai, Hiu-Fung Yuen, Shu-Dong Zhang, Youzhi Zhu, Yao Lin, Hang Fai Kwok

**Affiliations:** 1 Faculty of Health Sciences, University of Macau, Avenida de Universidade, Taipa, Macau, China; ravikiran.mahadevappa@gu.se (R.M.); hneveslab@gmail.com (H.N.); anneyuen2001@gmail.com (S.M.Y.); yb77607@umac.mo (M.J.); yuchenb@student.unimelb.edu.au (Y.B.); 2 Institute of Molecular and Cell Biology, A*STAR, Singapore 138673, Singapore; hfyuen@imcb.a-star.edu.sg; 3 Northern Ireland Centre for Stratified Medicine, Biomedical Sciences Research Institute, Ulster University, Londonderry BT47 6SB, UK; sd.zhang@ulster.ac.uk; 4 Department of Thyroid and Breast Surgery, The First Affiliated Hospital of Fujian Medical University, Fuzhou 350005, China; zhu1755@yeah.net; 5 Provincial University Key Laboratory of Cellular Stress Response and Metabolic Regulation, College of Life Sciences, Fujian Normal University, Fuzhou 350117, China; yaolin@fjnu.edu.cn

**Keywords:** MCM10, breast cancer, knockdown, overexpression, proliferation, survival

## Abstract

Breast cancer is one of the most common malignancies in women worldwide. In breast cancer, the cell proliferation rate is known to influence the cancer malignancy. Recent studies have shown that DNA replication initiation/licensing factors are involved in cancer cell proliferation as well as cancer cell migration and invasion. Licensing factors have also been reported as important prognostic markers in lung, prostrate, and bladder cancers. Here, we studied the role of MCM10, a novel licensing factor, in breast cancer progression. From the public database, NCBI, we investigated six independent breast cancer patient cohorts, totaling 1283 patients. We observed a significant association between high MCM10 mRNA expression with tumor grading and patients’ survival time. Most importantly, using breast cancer cohorts with available treatment information, we also demonstrated that a high level of MCM10 is associated with a better response to conventional treatment. Similarly, in in vitro studies, the expression level of MCM10 in breast cancer cell lines is significantly higher compared to paired normal breast epithelium cells. Knockdown of MCM10 expression in the cancer cell line showed significantly decreased tumorigenic properties such as cell proliferation, migration and anchorage independence. The MCF7 breast cancer cell line, after MCM10 expression knockdown, showed significantly decreased tumorigenic properties such as cell proliferation, migration, and anchorage independent growth. Mechanistically, MCM10 expression is observed to be regulated by an Estrogen Receptor (ER) signaling pathway, where its expression is suppressed by the inhibition of the ER or serum withdrawal. Our results suggest that MCM10 plays an important role in breast cancer progression and is a potential prognostic/predictive biomarker and therapeutic target for breast cancer patients.

## 1. Introduction

Breast cancer is the second major cause of cancer related mortality in women, after lung cancer, worldwide [1]. An increased cell proliferation rate is among the few key capabilities acquired by cancerous cells. In normal cells, cell proliferation is tightly regulated by the binding of DNA licensing factors such as CDC6, Cdt1 and Minichromosome Maintenance proteins (MCM) proteins (MCM2-7) onto DNA replication initiation sites. We, along with others, have previously observed the high expression of CDC6, CDT1 and MCM2-7 proteins in breast cancer progression [2,3,4]. Alterations in the expression profiles of the licensing proteins have been linked to various aspects of cancer [5,6] such as cancer cell proliferation [7,8] and cancer invasion [9]. High expression of CDC6, CDT1 and MCM family proteins may initiate replication at multiple regions on the DNA, thereby facilitating cancer cell proliferation. Depletion of some of these licensing factors, in vitro, is known to reduce cell proliferation as well as induce programmed cell death [10]. Therefore, DNA licensing factors have been reported as important prognostic markers in cancers such as lung, prostrate, and bladder cancers [2]. Reduction of MCM proteins in cancer cells is known to induce sensitivity to drugs such as aphidicolin, camptothecin, hydroxyurea, etc., and thereby promote an anti-proliferative effect [11,12]. Hence, DNA licensing factors may be a potential target for future chemotherapy or for combinatorial therapy against cancer.

During replication, MCM10, a subtype of the MCM family, is essential for replication origin firing [13]. MCM10 along with other proliferative markers initiate DNA replication in late G1 or early S phase of cell cycle, thereby mediating cell proliferation. MCM10 protein is also known to recruit DNA polymerase-α as well as regulate its catalytic subunit, preventing the elongation of the damaged DNA [14,15]. Depletion of MCM10 has been observed to regulate the turnover of DNA polymerase-α [15]. MCM10 is also known to interact with other replication protein complexes such as the MCM2-7 complex, Replication protein A and Proliferating cell nuclear antigen (PCNA), thereby maintaining a steady state during DNA replication [2]. Consequently, dysregulation of MCM10 protein may lead to genomic instability and replication stress, which can, in turn, induce tumorigenesis [16]. MCM10 was shown to be upregulated in a neuroblastoma cell line [17], overexpressed in pancreatic, cervical, esophageal and urothelial cancers [18,19,20,21] and mutated in early gastric cancer specimens [22]. In cervical and urothelial cancers, high expression of MCM10 has been associated with a higher grade cancer or malignant cancer [18,19]. MCM10 was determined as one of the top-ranked genes that is enriched in cancer-associated pathways [23]. High MCM10 can initiate multiple DNA replication origins along with origins that serve as a backup origin on DNA [24]. This could shorten replication time and promote DNA mismatch, thereby promoting genomic instability. For instance, altered initiation of replication especially during DNA damage response could lead to carcinogenic event [25]. Alterations in replication timing and progression (replication stress) may drive the malignancy in cancer cells thereby lower survival of cancer patients. Although the exact mechanism on regulation of MCM10 is still unknown, high expression of MCM10 can serve as an indicator of cancer progression.

In the present study, we focused on understanding the importance of MCM10 and its regulation during breast cancer progression by using publicly available breast cancer patient cohorts, in vitro cell line experiments and in vivo mouse models.

## 2. Results

### 2.1. Association between MCM10 Expression and Tumor Grade

**Figure 1:**
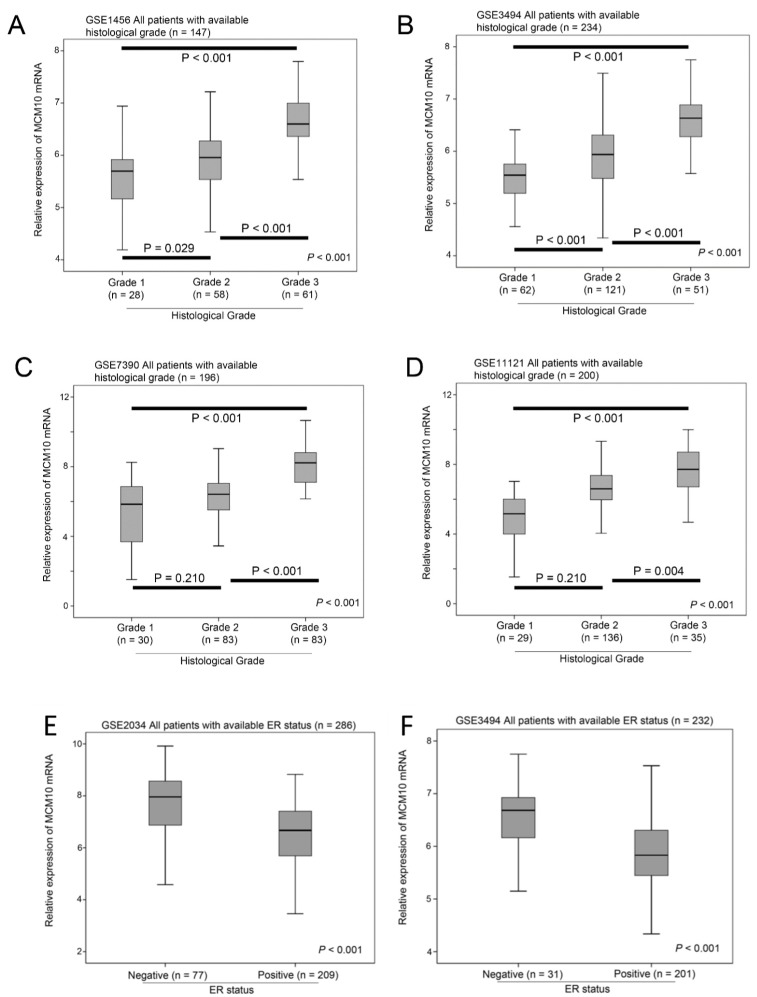

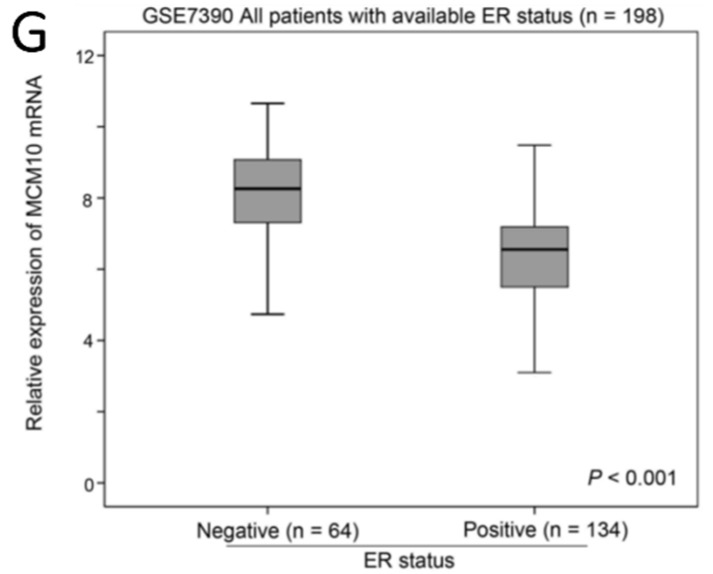
The association between MCM10 expressions, tumor grade and estrogen receptor status. Box plots showing the mean, 95% confidence interval and range of MCM10 mRNA expression in breast cancer datasets in tumors with different histologic grade in (**A**) GSE1456 (*n* = 147); (**B**) GSE3494 (*n* = 234); (**C**) GSE 7390 (*n* = 196) and (**D**) GSE11121 (*n* = 200); the association between MCM10 expressions and estrogen receptor status. Box plots showing the mean, 95% confidence interval and range of MCM10 mRNA expression in breast cancer datasets in tumors with different estrogen receptor status in (**E**) GSE2034 (*n* = 286); (**F**) GSE3494 (*n* = 232) and (**G**) GSE7390 (*n* = 198).

Six independent breast cancer patient cohorts, which we have used for studying MCM2-7 genes previously [4] using the Affymetrix platform, were included in the present study. Four out of the six independent breast cancer patient datasets have available information on tumor grading; therefore, we compared the expression level of MCM10 mRNA between tumors with different grades in these four datasets. MCM10 mRNA expression was consistently and significantly higher in tumors with a higher grade, in GSE1456 (*n* = 147, *p* < 0.001; Figure 1A), GSE3494 (*n* = 234, *p* < 0.001; Figure 1B), GSE7390 (*n* = 198, *p* < 0.001; Figure 1C) and GSE11121 (*n* = 200, *p* < 0.001; Figure 1D). In addition, three out of the six independent breast cancer patient datasets had available information on Estrogen Receptor (ER) status. We also compared the expression level of MCM10 mRNA between tumors with different ER statuses. MCM10 mRNA expression was consistently and significantly higher in ER negative breast cancer compared to ER positive breast cancer in GSE2034 (*n* = 286, *p* < 0.001; Figure 1E), GSE3494 (*n* = 232, *p* < 0.001; Figure 1F) and GSE7390 (*n* = 198, *p* < 0.001; Figure 1G). Our results suggest that MCM10 expression is regulated by ER signalling. Since the availability of data varies among the datasets included in the current study, we also analysed whether MCM10 expression is an independent prognostic marker in different conditions (Table 1). We have shown, by using Cox-regression analysis, that MCM10 expression predicted disease-specific events independent of histological grade (Supplementary Figure S1; MCM10: Hazard Ratio = 1.908, 95% CI = 1.362–2.671, *p* < 0.001; Grade: *p* = 0.048) in the combined dataset and independent of subtype (Supplementary Figure S2; MCM10: Hazard Ratio = 4.658, 95% CI = 2.132–10.179, *p* < 0.001).

### 2.2. Association between MCM10 Expression and Patient Survival

**Figure 2:**
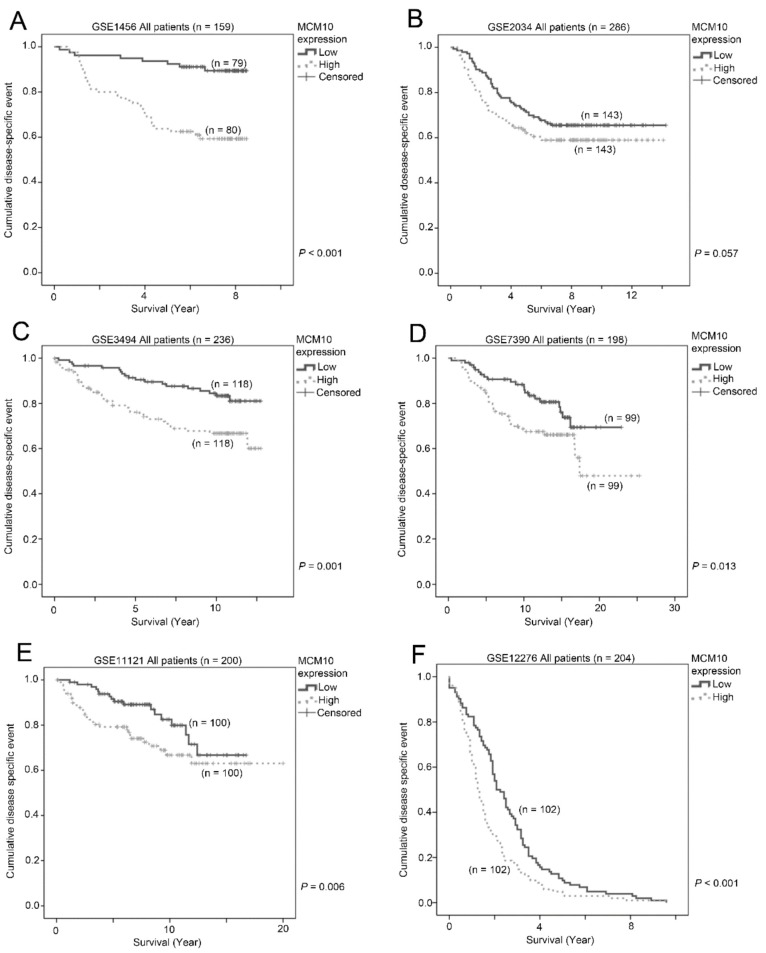
The association between MCM10 expressions and patient survival. Kaplan-Meier plots showing the proportion of patient survival for those with low or high MCM10 expression levels in breast cancer datasets (**A**) GSE1456 (*n* = 159); (**B**) GSE2034 (*n* = 286); (**C**) GSE3494 (*n* = 236); (**D**) GSE7390 (*n* = 198); (**E**) GSE11121 (*n* = 200); and (**F**) GSE12276 (*n* = 204).

A high expression level of MCM10 in tumor specimens was associated with a shorter survival time in all six breast cancer patient datasets tested (*n* = 1283). In GSE1456 (*n* = 159), patients with a high MCM10 expression level had a mean survival time of 6.2 years (95% CI = 5.5–6.8 years) versus 7.9 years (95% CI = 7.6–8.3 years) for those with a low MCM10 expression level (*p* < 0.001; Figure 2A). In GSE2034 (*n* = 286), patients with a high MCM10 expression level had a mean survival time of 9.2 years (95% CI = 8.3–10.2 years), while those patients with a low MCM10 expression level had a mean survival time of 10.4 years (95% CI = 9.5–11.3 years, *p* = 0.057; Figure 2B). In GSE3494 (*n* = 236), patients with a high MCM10 expression level had a mean survival time of 9.6 years (95% CI = 8.7–10.5 years), while those with a low MCM10 expression level had a mean survival time of 11.4 years (95% CI = 10.9–12.0 years, *p* = 0.001; Figure 2C). In GSE7390 (*n* = 198), patients with a high MCM10 expression level had a mean survival time of 17.0 years (95% CI = 14.8–19.3 years), while those with a low MCM10 expression level had a mean survival time of 19.0 years (95% CI = 17.5–20.5 years, *p* = 0.013; Figure 2D). Similar results were obtained from GSE11121, where patients with tumors expressing low MCM10 levels had a significantly longer survival compared to those with tumors expressing high MCM10 levels (*p* = 0.006; Figure 2E). For those patients with metastatic breast cancer, from GSE12276 (*n* = 204), the median survival for patients with a high MCM10 expression level was only 1.3 years and 2.1 years for those with a low MCM10 expression level (*p* < 0.001; Figure 2F). Summary of the survival analysis in breast cancer patient datasets is attached in (Table 2). Since the observations were consistent among all six independent breast cancer patient datasets, our results strongly suggest that MCM10 alone could be a prognostic marker for breast cancer patients.

### 2.3. MCM10 Protein Level Was Increased in Human Breast Cancer Specimens

**Figure 3:**
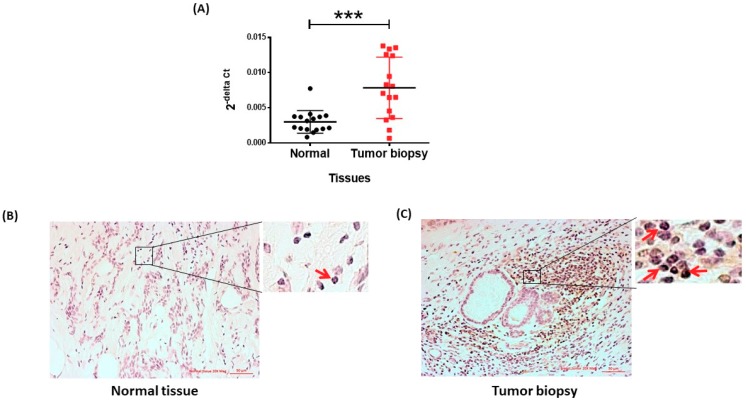
MCM10 expression in patient tumor biopsy. (**A**) MCM10 expression quantified by qPCR in paired normal and tumor biopsy samples (*n* = 16, *** *p* = 0.006); (**B**,**C**) immunohistochemistry staining of patients’ biopsy, High MCM10 DAB staining is observed in patients’ biopsies; (**C**) compared to normal breast tissue (**B**). Magnified images showing nuclear staining of MCM10, in red arrows.

To validate our observation from the publicly available breast cancer patient datasets, we analyzed the expression of MCM10 by qPCR and immunohistochemistry from 16 pairs of normal and tumor samples obtained from breast cancer patients having a biopsy in the First Affiliated Hospital of Fujian Medical University. MCM10 mRNA expression was found to be significantly higher (*p* = 0.006) in tumor samples compared to the respective normal tissue. (Figure 3A) The results didn’t correlate to any of clinicopathological details of patients (Supplementary Table S1). Similarly, protein expression of MCM10 was significantly higher in tumor specimens compared to its normal breast epithelium counterparts (Figure 3B,C), confirming our observation using microarray data in six independent breast cancer patient cohorts.

### 2.4. MCM10 Expression Was Increased in Breast Cancer Cell Lines

**Figure 4:**
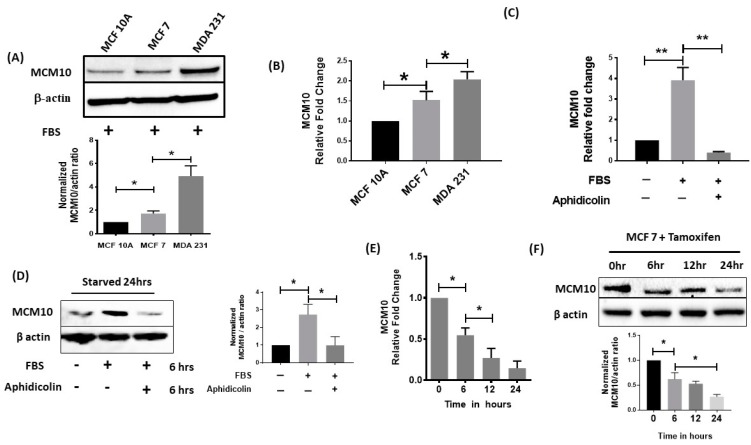
Relative expression of MCM10 in different cells in vitro. (**A**,**B**) relative protein quantification by Western blots and qPCR for the normal breast epithelial cell line MCF10A and for the breast cancer cell lines MCF 7 and MDA 231. High expression of MCM10 was found in MDA 231 cells, which are the most aggressive cells. (*n* = 3, * *p* < 0.05) (**C**,**D**) expression of MCM10 under stress induced by Fetal bovine serum (FBS) starvation in MDA231 cells. Starved MDA 231 cells showed an increase in MCM10 protein level after six hours in FBS. Interestingly, addition of proliferation inhibitor Aphidicolin further blocked expression of MCM10 observed by Western blots as well as qPCR. (*n* = 3, ** *p* < 0.01) (**E**,**F**) Tamoxifen treatment to MCF 7 cells reduced MCM10 expression in a time dependent manner observed by qPCR and Western blots. (*n* = 3, * *p* < 0.05).

We further studied the expression of MCM10 in breast cancer cell lines. MCF 10A is a normal breast cell line that we used to compare with two different breast cancer cell lines, MCF 7 (ER-positive; less aggressive) and MDA 231 (triple negative; more aggressive). We found a significantly increased expression of MCM10 in both MCF 7 and MDA 231 cells compared to MCF 10A cells. Furthermore, MCM10 expression was also significantly higher in MDA 231 cells when compared to MCF-7 cells using both the protein and mRNA levels for comparison (Figure 4A,B).

### 2.5. MCM10 Expression Was Regulated by Growth Signalling

To investigate whether MCM10 expression is regulated by a proliferation signal, MDA 231 cells were incubated in media with or without serum for 24 h, and the expression of MCM10 was tested. We observed a significant increase in MCM10 expression level both in qPCR and Western blot for MDA 231 cells grown in media containing serum compared to the cells that grew in media without serum. However, the increase in MCM10 expression from the addition of serum was not observed when the cells were treated with aphidicolin, which blocked cell cycle progression, indicating that MCM10 expression is associated with cell proliferation in breast cancer cells (Figure 4C,D). ER signaling is an important growth signal for breast cancer. While there was a differential expression of MCM10 in ER positive MCF-7 cells compared to ER negative MDA-MB-231 cells, we further investigated whether MCM10 expression is regulated by ER signaling. We found that, when ER positive MCF-7 cells were treated with tamoxifen, the mRNA and protein expression of MCM10 decreased starting at six hours and lasted 24 h post-treatment (Figure 4E,F).

### 2.6. Knockdown of MCM10 Decreased Cell Proliferation, Cell Migration and Anchorage Independent Growth

**Figure 5:**
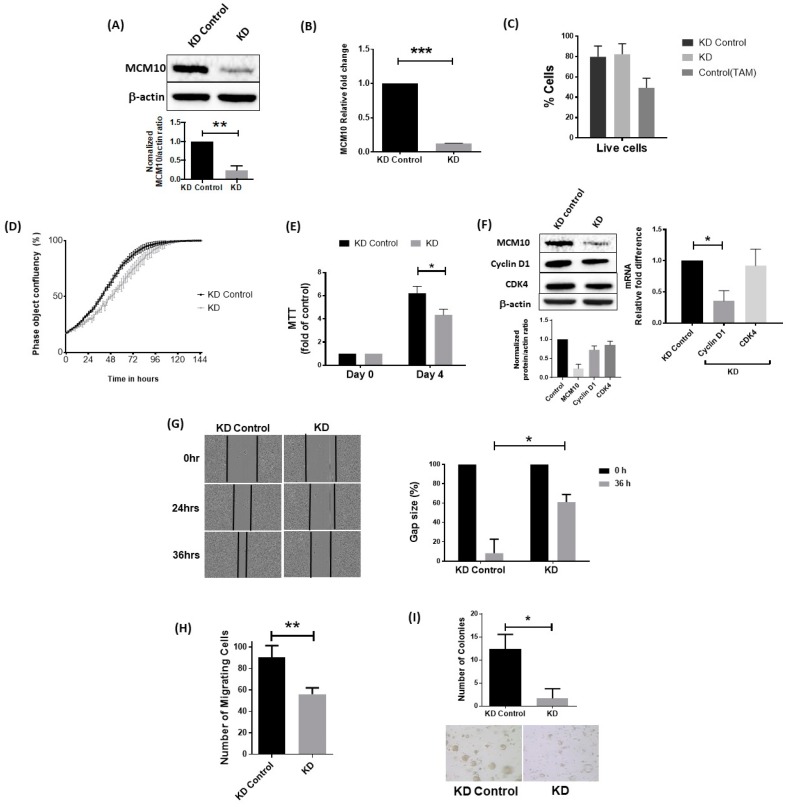
Cancer hallmarks of stable MCM10 knockdown (KD) in MCF 7 cells. (**A**,**B**) relative expression of MCM10 in stable MCM10-KD MCF 7 cell lines assessed by qPCR and Western blots (*n* = 3, *** *p* < 0.001); (**C**) apoptosis analysis by Annexin V/Propidium Iodide flow cytometry showed no difference between stable MCM10-control MCF7 cell lines and stable MCM10-KD MCF 7 cell lines (*n* = 3, * *p* < 0.05); (**D**) following MCM10 KD, the MCM10-KD MCF7 cells showed a decrease in cell proliferation monitored for seven days using phase contrast images in Incucyte ZOOM analysis software (*n* = 6); (**E**) MTT assay using stable cells showed a similar observation indicating decreased proliferation in MCM10-KD MCF 7 cells (*n* = 3, * *p* < 0.05); (**F**) analysis cell cycle markers by western blot and QPCR showed a decrease in cycling D1 expression indicating impaired cell proliferation in in MCM10-KD MCF 7 cells (*n* = 3, * *p* < 0.05); (**G**) wound healing migration assay performed by using Incucyte ZOOM showed a decrease in cell migration in MCM10-KD MCF 7 cell lines. Wound heal quantified by relative Gap size using Incucyte ZOOM software (*n* = 6, * *p* < 0.05); (**H**) Transwell cell migration assay was used to confirm our observation and showed a similar decrease in the number of migrating cells in MCM10-KD MCF 7 cells (*n* = 3, ** *p* < 0.01); (**I**) colony formation assay performed using soft agar showed a decrease in the number of colonies in MCM10-KD MCF 7 cells compared to the control cells. (*n* = 3, * *p* < 0.05).

To investigate how MCM10 may contribute to breast cancer progression, we knocked down MCM10 expression in MCF 7 cells and then tested the cellular properties, including cell proliferation and migration. As shown in Figure 5A,B, knockdown of MCM10 resulted in decreased expression of MCM10 at both the mRNA and protein levels. Knockdown of MCM10 did not increase apoptosis in MCF 7 cells compared to the control cells, suggesting that MCM10 is not essential for cell survival (Figure 5C). On the other hand, MCM10 knockdown MCF 7 cells had a significantly lower proliferation rate compared to the control MCF 7 cells (Figure 5D,E). Indeed, we also observed a significant reduction in cyclin D1 expression in the MCM10 knockdown MCF 7 cells, suggesting cell proliferation was impaired when MCM10 expression was knocked down (Figure 5F). Similarly, MCM10 knockdown MCF-7 cells also had a significantly lower migration and soft agar colony formation potential, suggesting that reducing the expression of MCM10 may reduce the aggressiveness of breast cancer cells (Figure 5G–I). However, further increasing the level of MCM10 by ectopic expression did not further increase cell proliferation and migration in MCF-7 cells (Supplementary Figure S3). Overexpression of MCM10 in MCF 7 cells did not reverse our earlier observation in MCM10 KD cells (Supplementary Figure S3B–E). Overexpression monitored by FLAG tag expression showed that the level of MCM10 proteins was indeed maintained in steady state by unknown regulatory mechanism in MCF 7 cells.

### 2.7. MCM10 Expression Correlated with Tumor Growth In Vivo 

**Figure 6:**
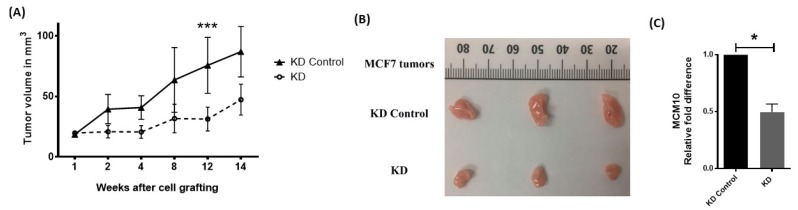
Effect of MCM10 knockdown in tumor progression in vivo. (**A**) tumor growth monitored by tumor volume measurement and showed a significant difference at the 12th week after grafting MCM10-KD MCF7 cell (*n* = 12, *** *p* < 0.001); (**B**) representative tumors at 14 weeks after grafting; (**C**) relative quantification of MCM10 in mice tumors (*n* = 3, * *p* < 0.05).

To further understand the effect of MCM10 knockdown on tumor growth in vivo, we grafted MCF 7 knockdown cells into the mammary fat pad of nude mice. Tumor volume was measured weekly, and all mice were sacrificed at the end of experiment when tumors were dissected and weighed. The inhibitory effect of MCM10 knockdown on the growth of breast tumors was significant in xenograft model mice. Tumor growth was slower and a significant difference was observed on the 12th week after cell grafting (Figure 6A,B). We isolated the tumor on the 14th week and analyzed expression of MCM10 using qPCR (Figure 6C). The tumors formed by MCM10 knockdown cells showed a significant decrease in MCM10 expression compared to the control cells, suggesting that reduction of MCM10 also reduced tumor growth in vivo in mice models.

### 2.8. Association between MCM10 Expression and Treatment Response

**Figure 7:**
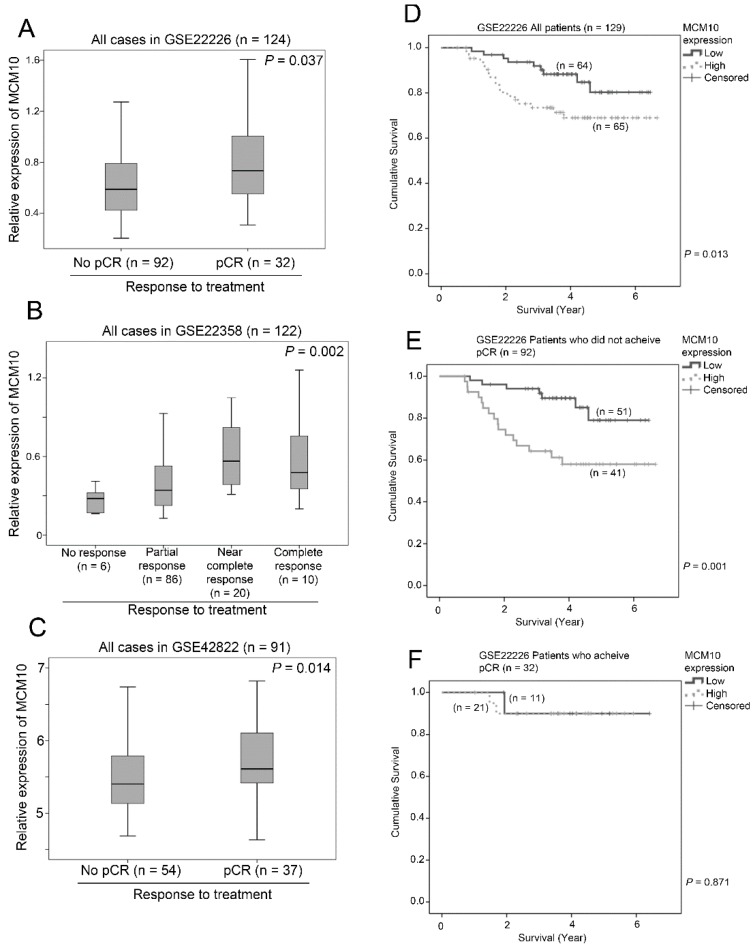
The association between MCM10 expression and response to neoadjuvant chemotherapy. Box plots showing the mean, 95% confidence interval and range of MCM10 mRNA expression in breast cancer datasets in patients with various response to chemotherapy in (**A**) GSE 22226 (*n* = 124); (**B**) GSE22358 (*n* = 122) and (**C**) GSE42822 (*n* = 91). Kaplan-Meier plots showing proportion of patients survived for those with low or high level expression of MCM10 in GSE22226; (**D**) the whole patient cohort; (**E**) patients who did not achieve pCR and (**F**) patients who achieved pCR.

We went on to investigate whether expression of MCM10 was associated with response to treatment. Three independent breast cancer patient datasets with treatment details (chemotherapy with or without trastuzumab depending on the HER2 status) and response to treatment were included in the analysis. In GSE22226 (*n* = 124), patients who achieved pathological complete response (pCR) after neoadjuvant treatment had a significantly higher expression of MCM10 compared to those patients who did not achieve pCR after treatment (*p* = 0.037; Figure 7A). In GSE22358 (*n* = 122), patients who achieved near complete response or complete response had a higher level expression of MCM10 compared to those patients who had only partial response or no response to the neoadjuvant treatment (*p* = 0.002; Figure 7B). Similarly, in GSE42822 (*n* = 91), patients who achieved pCR had a significantly higher expression of MCM10 compared to those who did not achieve pCR (*p* = 0.014; Figure 7C). These consistent results obtained in three independent breast cancer datasets suggest that tumors with higher MCM10 expression may be more prompt to respond to neoadjuvant treatment. Further to this observation, we found that MCM10 expression was associated with survival, especially in those patients who did not achieve a pCR during neoadjuvant treatment. Similar to the other six independent breast cancer datasets, in GSE22226, even with standard neoadjuvant treatment, patients with a high level expression of MCM10 had a significantly shorter survival time compared to those with a low level expression of MCM10 (*p* = 0.013; Figure 7D). Importantly, in patients who did not achieve pCR, a high level expression of MCM10 was associated with a shorter survival time (*p* = 0.001; Figure 7E), while, in patients who achieved pCR, there was no significant association between MCM10 expression and survival (*p* = 0.871; Figure 7F). These results suggest that MCM10 could be a potential prognostic marker to stratify which patients should require more intensive adjuvant treatment when pCR could not be achieved via neoadjuvant treatment.

## 3. Discussion

DNA replication is a highly regulated process in the cell. The MCM protein complex plays a vital role as DNA helicases during replication. Excess MCM proteins are known to maintain genomic integrity by acting as dormant origins that serve as a backup during replication [24]. MCM10 initiates DNA replication by activating MCM replication complex, which implies that its dysregulation may contribute to uncontrollable proliferation, thereby, leading to cancer development [18,19,20,21]. However, to our best knowledge, no report has extensively investigated the significance of MCM10 in breast cancer. In the present study, we have demonstrated that (i) MCM10 was highly expressed in human breast cancer specimens compared to normal breast epithelium, in vitro and (ii) a high MCM10 expression level was associated with a significantly shorter time of breast cancer patient survival. We demonstrated that (iii) MCM10 expression is required for cell proliferation and migration in ER-positive breast cancer cells, in which knockdown of MCM10 resulted in reduction in these cellular properties. These results indicate a positive correlation between DNA replication and breast cancer cell proliferation. These results also suggest a use of MCM10 as a prognostic marker as well as a potential therapeutic target for breast cancer.

In total, six independent breast cancer patient cohorts with 1283 individual patients were included in the present study for the survival analysis; the robust results from these six independent cohorts suggest that the association between MCM10 expression and patient survival was consistent. Since MCM10 expression is associated with cell cycle progression and cell proliferation [32,33,34], it is not surprising that tumors with high MCM10 expression levels responded to chemotherapy that targets fast growing cells. Consistently, observed in three independent datasets, breast cancer patients who achieved pCR by neoadjuvant chemotherapy had a significantly higher MCM10 expression level. Another important observation in this study is that patients who had low MCM10 expression level and achieved pCR by neoadjuvant therapy could survive significantly longer than those patients who achieved pCR but had a high MCM10 expression level, suggesting that patients with a high expression level of MCM10 may have a greater chance of recurrence even achieving pCR during neoadjuvant therapy.

Loss of MCM proteins has previously been reported to reduce proliferation of cancer cells [11,35]. Serum deprivation itself induced a significant reduction in MCM10 expression as well as cell proliferation. Knockdown of MCM10 in ER positive breast cancer cells also reduced cell proliferation rate and cancer migration. Reduction of cancer cell properties after MCM10 knockdown in ER positive breast cancer cells was in parallel to three independent ER-positive breast cancer patient cohorts with less expression of MCM10 and longer survival time. In addition, these MCM10 knockdown cells had a slower growth rate compared to control cells in vivo, suggesting that MCM10 plays an important role in cancer-related properties in breast cancer cells. Overexpression of MCM10 in MCF 7 cells (Supplementary Figure S1), monitored by stable expression of FLAG tag, did not affect the proliferation rate or any of the key hallmarks of cancers. This observation indicates presence of a regulatory mechanism that maintains a steady state of MCM10 within the cell. Hence, studies on post-translational regulation of MCM10 are required to analyze the activation and initiation mechanism of MCM10 during cancer. However, the potential utility of reducing MCM10 in cancer therapy is not clear; nonetheless, it can decrease initiation of DNA replication. MCM10 downregulation can block dormant DNA replication origins that serve as backup during replication [24]. Targeting MCM10 also plays an important role when using anti-cancer agents such as platinum based drugs, 5-fluoro uracil, etc., which bind to DNA forming DNA lesions. Thus, targeting MCM10 can increase the therapeutic potential of conventional drugs. Reduction of MCM proteins in cancer cells is also known to induce sensitivity to drugs that block replication fork progression such as aphidicolin, camptothecin, hydroxyurea, etc., and thereby promoting anti-proliferative effect [11,12].

Estrogen receptors play an important role in breast cancer development and progression. MCM2, MCM6 and MCM7 have been shown to be regulated by ER signaling [26,36,37]. ER signaling was also associated with an increase in MCM10 expression in vivo [37]. In female mice, treatment of estradiol induced increased proliferation of natural killer cells by upregulating MCM7 and MCM10 expressions [37]. Using the ER positive MCF 7 cells, we found that the blockade of the ER signaling by tamoxifen resulted in a significant reduction in MCM10 expression. These results suggest that MCM10 is a downstream factor of ER signaling in breast cancer cells. However, stimulation using estradiol showed perplexing results. In parallel to this observation, in ER-negative breast cancer cohorts and also in ER-negative MDA MB 231 cells, MCM10 expression was significantly higher compared to ER-positive specimens/cells. The previous evidence also indicates differential regulation of MCM10. Studies indicate that ER-negative breast cancer cells seems to have extensive deregulations in their estrogen receptor pathway since restored ER expression in ER-negative breast cancer cells did not restore the ability of estrogen to stimulate proliferation [27]. Nonetheless, the regulation of MCM10 may be vital for both ER-positive and ER-negative breast cancer cells, which needs further investigation.

## 4. Materials and Methods

Ethics approval and consent to participate: all animal handling was approved by the University of Macau Animal Ethics Committee (UMARE-012-2016). Study participants signed an informed consent to allow research assays to be carried out on their tumor tissue. A Medical Ethics Committee at the First Affiliated Hospital of Fujian Medical University approved the study ([2015]108).

### 4.1. Extraction of Clinical and Microarray Gene Expression Data from Breast Cancer Patient Datasets

Six independent breast cancer patient datasets, GSE1456 [28], GSE2034 [29], GSE3494 [30], GSE7390 [31], GSE11121 [38] and GSE12276 [39], each comprising 150 or more patients, with available data on patient survival, clinicopathological parameters and MCM10 gene expression in the Gene Expression Omnibus (GEO) Database were included in this study, while three independent breast cancer patient datasets, each comprised of 80 or more patients, GSE22226 [40], GSE22358 [41] and GSE42822 [42], with available information on response to treatment, hormonal and HER2 receptor status were also included. Microarray gene expression data were retrieved from the data matrixes deposited to the GEO database by the original authors. R scripting was used to extract the expression values of genes (probesets) of interest and the clinical data from the data matrixes were downloaded from GEO.

### 4.2. Correlations of Gene Expression Levels and Clinical Data

All statistical analyses were performed using SPSS19.0 (IBM Corp., Armonk, NY, USA). The associations between expression level of MCM10 and tumor grade, between expression level of MCM10 and ER-status, between expression level of MCM10 and treatment response, and between expression level of MCM10 and triple negative status were tested by ANOVA or Welch’s *t*-test where applicable depending on the *p*-value of homogeneity test. Expression levels were divided into high and low levels using median expression level as the cut-off point for Kaplan–Meier survival analysis. Results were compared by a Wilcoxon–Gehen test.

### 4.3. Cell Culture and Reagents

All cell lines were obtained commercially from ATCC (American Type Culture Collection, Manassas, VA, USA). MCF10A were maintained in Dulbecco’s Modified Eagle Medium: Nutrient Mixture F-12 (DMEM/F12) (Gibco, Carlsbad, CA, USA) supplemented with 5% horse serum, 20 ng/mL EGF (Peprotech, Rocky Hill, NJ, USA), 0.5 µg/mL hydrocortisone (Sigma-Aldrich, St. Louis, MO, USA), 100 ng/mL cholera toxin (Sigma-Aldrich) and 10 µg/mL insulin (Sigma-Aldrich). MCF7 were maintained in Dulbecco’s Modified Eagle Medium (DMEM) (Gibco) with 10% FBS, 1% sodium pyruvate (Gibco), 1% L-glutamine and 10 µg/mL insulin (Sigma-Aldrich). MDA-MB-231 were maintained in DMEM (Gibco) supplemented with 10% FBS, 1% sodium pyruvate (Gibco), and 1% L-glutamine. HEK-293T cells (provided by Dr Joong Sup SHIM, University of Macau, Macau SAR, China) and Phoenix-AMPHO (purchased from ATCC) for lentiviral and retroviral packaging, respectively, were maintained in DMEM supplemented with 10% FCS. The medium for all cell lines was supplemented with 1% Pen/ Strep (Gibco) except when otherwise noted. All cells were cultured in an incubator with a humidified atmosphere maintained at 5% CO_2_ and 95% air at 37 °C. For the soft agar, migration and invasion assays, phenol red-free DMEM was used. All cells were free of mycoplasma contamination.

### 4.4. Transfection

*MCM10* expression was silenced in MCF7 cells using specific shRNA (TRCN0000245427: 5′CCGGAGATGCAGGAGCGCTACTTTGCTCGAGCAAAGTAGCGCTCCTGCATCTTTTTTG3′) (Sigma) against *MCM10*. Lentiviruses were packaged using expression plasmid, constructed using expression vectors: pCI-VSVG (#1733) and pCMV-dR8.2 dvpr (#8455) (Addgene, Cambridge, MA, USA) along with the shRNA using Lipofectamine 2000 according to manufacturer’s instructions. The lentivruses were produced and delivered to the MCF-7 cells to create a stable *MCM10* knockdown cell line.

To upregulate *MCM10* expression in MCF7 cells, retroviruses were first prepared using expression vectors containing pBabe-puro-MCM10-3Xflag or expression vectors alone (both vectors were obtained from Lin Yao from Fujian Normal University, Fujian, China) and transfected into Phoenix-AMPHO cells using Lipofectamine 3000, according to the manufacturer’s instructions. The retroviruses were then introduced to MCF7 cells to create a stable *MCM10* overexpression cell line. All constructions used were sequenced and confirmed to be correct.

### 4.5. Quantitative qPCR

MCF10A, MCF7 and MDA-MB-231 cells were seeded at a density of 2 × 10^5^ cells/well on 6-well plates. Cells were then harvested by cell scraping in RLT lysis buffer and total RNA was isolated using RNeasy Mini Kit and QiaShredder (Qiagen, Valencia, CA, USA) according to the manufacturer’s instructions. RNA quantity and quality were measured using Nanodrop™ spectrophotometer (NanoDrop Technologies, Waltham, MA, USA). First strand cDNA synthesis was performed from 1 μg total RNA using High Capacity cDNA Reverse Transcription Kit (Applied Biosystems, Waltham, MA, USA) on a BioRad C1000 Touch™ Thermal Cycler (BioRad, Hercules, CA, USA). qRT-PCR analysis was performed using the M×3005P qPCR System (Agilent, Santa Clara, CA, USA) and TaqMan^®^ Universal PCR Master Mix (Applied Biosystems) and Taqman probes specific for mcm10 and house-keeping gene glyceraldehyde 3-phosphate dehydrogenase (GAPDH). The Taqman probes (mcm10, Hs00960349_m1 and GAPDH, Hs02758991_g1) were purchased from ThermoFisher Scientific (Waltham, MA, USA). For Cyclin D1 and CDK4 analysis, we used in-house designed primers. Fold change in gene expression was normalized to GAPDH and compared to the untreated value using the 2^−ΔΔCT^ formula.

### 4.6. Western Blotting

Cells were seeded in T25 flask, harvested using cell scrapers and suspended in RIPA buffer (150 mM NaCl; 5mM EDTA, 50 mM Tris, 1% Triton X-100, 0.5% sodium deoxycholate, 0.1% SDS) supplemented with protease inhibitor (Roche, St. Louis, MO, USA) and phosphatase inhibitor (Roche). Protein samples were incubated for 30 min at 4 °C with intermittent agitation after which they were centrifuged for purification and quantified using BCA protein assay (ThermoFisher Scientific). The proteins were separated by 4–12% SDS-polyacrylamide gel electrophoresis (SDS-PAGE) and electrophoretically transferred to a nitrocellulose membrane (Millipore, Hercules, CA, USA) on a Mini Trans-Blot Electrophoretic Transfer Cell (BioRad, Hercules, CA, USA). The membrane containing the transferred protein was blocked with 3% non-fat milk at room temperature for an hour. Target proteins were detected by incubating the membrane at 4 °C overnight with primary anti-MCM10 antibody (Bethyl, Montgomery, TX, USA) (1:1000), anti-Cyclin D1 antibody (#2922 Cell Signaling Technology, Beverly, MA, USA) (1:1000), anti-CDK4 antibody (#12790 Cell Signaling technology) (1:1000), anti-FLAG antibody (#F1804-5MG Sigma Aldrich, Taufkirchen, Germany) (1:5000) and primary anti-β-actin antibody (Santa Cruz Biotechnologies, Dallas, TX, USA) (1:5000) (1:5000). The appropriate horseradish peroxidase (HRP)-conjugated secondary antibodies (1:5000) (Santa Cruz Biotechnologies, Dallas, TX, USA) were applied to the membrane and incubated at 1 h at room temperature. Positive bands were detected using Immobilon Western Chemilum HRP substrate (Merck Millipore, Billerica, MA, USA). Blots were visualized on ChemiDoc Touch Imaging System (BioRad). Quantitation of immunoreactive signals was carried out by densitometry using ImageJ 1.46r software (NIH, Bethesda, MA, USA). The value of each band was normalized to β-actin.

### 4.7. Cell Proliferation by Incucyte Zoom

To monitor the proliferation rate, cells were seeded at 2.25 × 10^4^ cells per well in full growth media on 12-well, clear bottomed, tissue culture plates (ThermoFisher Scientific). The plate was then inserted into the IncuCyte (Essen Bioscience, Ann Arbor, MI, USA) for real-time imaging, with sixteen fields imaged per well under 10× magnification every 2 h for a total of 4 days. Data were analyzed using the IncuCyte Zoom software (version 2014a Essen Bioscience, Ann Arbor, MI, USA), which quantified cell surface area coverage as confluence values. All IncuCyte experiments were performed in triplicate.

### 4.8. MTT Assay

Cells were treated at 5 × 10^3^ on 96-well, clear bottomed, tissue culture plates (ThermoFisher Scientific) in 200 µL complete growth medium. The MTT assay was applied to study the cell viability after 96 h. Cells were rinsed once with PBS (Gibco), and incubated with serum-free medium containing 20 µL of 0.4 mg/mL MTT at 37 °C and 5% CO_2_ for 4 h. The MTT solution was then removed, and 150 µL of dimethylsulfoxide (Sigma) was added into each well, and the plates were shaken for 10 min. The optical densities of the supernatant were read at 555 nm using a microplate spectrophotometer (Spectra Max 340, Molecular Devices, San Jose, CA, USA).

### 4.9. Wound Healing Assay

Cells were assessed in wound healing scratch assays using the IncuCyte (Essen Bioscience). Cell migration assays were achieved by growing MCF7 and MDA-MB-231 cells to confluence in 96-well plates (Essen BioScience; cat. no. 4379). Wounds were made using the 96-pin wound-making tool (WoundMaker; Essen Bioscience) to simultaneously create a precise and reproducible wound in each well, 1 h after the plate was washed twice with PBS, and incubated with DMEM containing 1% FBS, 1% penicillin-streptomycin (Gibco), 4 mM L-glutamine (Gibco), sodium pyruvate (1 mM) and 0.01 mg/mL bovine insulin (Sigma). Cell migration was monitored in real time by IncuCyte, and wound width was measured by the IncuCyte software Zoom (version 2014a, Essen Bioscience, Ann Arbor, MI, USA).

### 4.10. Transwell Migration Assay

The migration ability of cells was detected using CytoSelect™ 24-Well Cell Migration (Cell Biolabs, San Diego, CA, USA) according to manufacturer’s instructions. Briefly, cells were starved overnight prior to the running of the assay. In addition, 1 × 10^6^ cells/well were seeded in the upper chamber with 300 µL of serum-free medium. The bottom chamber received 500 µL of full medium. After 24 h, the media from inside of the insert was aspirated and non-migratory cells were cleaned using cotton swabs. The inserts were then transferred to a clean well containing 400 µL of cell stain solution and incubated for 10 min at room temperature. The stained inserts were then washed a few times in a beaker of water and allowed to air dry and the migratory cells were counted under a light microscope (Carl Zeiss Axio Observer, Oberkochen, Germany).

### 4.11. Soft Agar Colony Formation Assay

Cells were collected and mixed at a density of 2.5 × 10^5^ cells mL^−1^ with 0.6% low melting point agarose (Lonza, Basel, Switzerland) in complete DMEM phenol red-free medium containing growth medium for a final concentration of 0.3% agarose. The cell mixture was plated on top of a solidified layer of 0.6% agarose in growth medium. Cells were fed every 3–4 days with growth medium. Colonies greater than 50 µm 22 days post-plating, were counted and photographed at 10× magnification under a microscope (Leica M165FC stereomicroscope, Buffalo Grove, IL, USA) and analyzed using ImageJ 1.46r software (NIH, Bethesda, MA, USA). At least two independent experiments were performed in triplicate.

### 4.12. Drug Assay

Cells were seeded at a density of 2 × 10^5^ cells/well in 6-well plates and allowed to attach overnight. MCF-7 cells were then treated with tamoxifen (20 μM, Sigma) and incubated at 37 °C in a humidified atmosphere with 5% CO_2_. Tamoxifen treatment cells were then collected at 0 h, 6 h, 12 h and 24 h for qPCR and Western blot analysis. To block cell proliferation, MDA 231 cells were treated with 25 μM Aphidicolin.

### 4.13. Assessment of Apoptosis

Apoptosis was investigated with the Alexa Fluor^®^ 488 Annexin V/Dead Cell Apoptosis Kit (ThermoFisher Scientific) as per the manufacturer’s instructions, on a BD Accuri C6 Flow Cytometer (BD Biosciences, Franklin Lakes, NJ, USA).

### 4.14. Immunohistology

Patient samples were obtained from breast cancer patients at the First Affiliated Hospital of Fujian Medical University with Medical Ethics Committee of the First Affiliated Hospital of Fujian Medical University approval (approval number: [2015]108, Date: January 2015). The samples were immersion fixed followed by dehydration in ethanol and embedded in paraffin wax. The tissues were cut, sectioned and mounted on SuperFrost Plus Slides (ThermoFisher Scientific). The sections were then de-paraffinized in xylene overnight and rehydrated in a stepwise ethanol gradient, followed by immersion in methanol containing 0.3% hydrogen peroxide and epitope retrieval with the Pre-treatment (PT) module (ThermoFisher Scientific) using TEG buffer. The sections were washed in 1% BSA and incubated overnight with the anti-MCM10 primary antibody at 1:250 dilution in 0.1% BSA. The sections were further washed with PBS and incubated with the HRP-conjugated secondary antibody (at a 1:500 dilution). Finally, the sections were developed using DAB (ZsBio, Beijing, China) and counterstained with Meiers Haematoxylin for 2 min followed by de-hydration in an ethanol gradient and xylene. Mounting was performed with Eukitt mounting medium (Merck) and imaging was achieved with Carl Zeiss Axio Imager 2 Microscope (Zeiss).

### 4.15. In Vivo Studies

All animal studies and ethics were followed the guidelines issued and approved by the University of Macau Animal Ethics Committee (UMARE-012-2016, Permission date: May 2016), respectively. Eighteen six-week female nude mice received subcutaneous injection with either stable MCF7-Knockdown cell line or cell line contain vector control. To maintain the tumor, a single dose of 50 μL PBS containing 3 μg estradiol was given on alternative days. The tumors were measured with calipers. Tumor volume was determined using the formula:

(1) $$\mathrm{Tumor}\;\mathrm{volume}=\frac{\pi }{6}\times L\times W^{2},$$

where L is the longest diameter and W is the shortest diameter of the excised tumor. At the end of the experiment, tumors were removed, weighed and photographed.

### 4.16. Statistical Analysis

The differences observed between the control and treated groups were analyzed using either One-way ANOVA or unpaired Student *t*-test (two-tailed) using GraphPad Prism 6 (GraphPad Software, La Jolla, CA, USA). The results were expressed as the Mean ± SEM (standard error of mean) from three different replicates and a value of *p* < 0.05 was considered statistically significant.

## 5. Conclusions

Our data demonstrated that MCM10 plays an important in breast cancer progression and is a potential prognostic biomarker as well as a therapeutic target for breast cancer patients.

## Figures and Tables

**Table 1:** Clinicopathological details of six different data sets used in the paper.

Dataset	Sample Size	Year of the Associated Publication	Available Clinical Parameters	Patient Characteristics (Percent)
GSE1456 [26]	159	2005	Grade	Grade 1 (18) Grade 2 (37) Grade 3 (38) Missing (7)
			Subtype	Luminal A (25) Luminal B (15) Basal (16) HER2 (9) Normal-like (23) Missing (13)
GSE2034 [27]	286	2005	ER status	ER negative (27) ER positive (73)
			Brain relapse status	With brain relapse (97) Without brain relapse (3)
GSE3494 [28]	251	2005	Grade	Grade 1 (27) Grade 2 (51) Grade 3 (22) Missing (1)
			ER status	ER negative (14) ER positive (85) Missing (2)
			PR status	PR negative (24) PR positive (76)
			Age at diagnosis	Mean = 62 year (SD = 14)
			Tumor size	Mean = 22 mm (SD = 13)
			Lymph node involvement	Negative (63) Positive (34) Missing (4)
GSE7390 [29]	198	2007	ER status	ER Negative (32) ER Positive (68)
			Grade	Grade 1 (15) Grade 2 (42) Grade 3 (42) Missing (1)
			Age	Mean = 46 (SD = 7)
			Tumor size	Mean = 22 mm (SD = 8)
GSE11121 [30]	200	2008	Grade	Grade 1 (15) Grade 2 (68) Grade 3 (18)
			Tumor size	Mean = 21 (SD = 10)
GSE12276 [31]	204	2009	Site of relapse	Local (9) Other (83) Brain (4) Brain and other (4)

Note: HER2, Human Epidermal Growth Factor Receptor 2; ER, Estrogen Receptor; PR, Progesterone Receptor.

**Table 2:** Summary of the survival analysis in breast cancer patient datasets.

Dataset	No. of Patients	*p*-Value
GSE1456	159	<0.001
GSE2034	286	0.057
GSE3494	236	0.001
GSE7390	198	0.013
GSE11121	200	0.006
GSE12276	204	<0.001

## Supplementary Materials

The following are available online at http://www.mdpi.com/2072-6694/10/9/282/s1, Figure S1: Breast cancer combined datasets with different histological grades, Figure S2: Breast cancer patient different subtypes, Figure S3: Relative expression of MCM10 in stable Overexpression (OE) in MCF 7 cell lines. Table S1: Clinicopathological parameters of patient samples used for qPCR analysis of MCM10.

## Abbreviations

DNA	Deoxyribonucleic acid
MCM	Minichromosome maintenance proteins
GEO	Gene Expression Omnibus
CDC6	Cell division cycle 6
CDT1	Chromatin licensing and DNA replication factor 1
PCNA	Proliferating cell nuclear antigen
HER2	Human Epidermal Growth Factor Receptor 2
GADPH	Glyceraldehyde 3-phosphate dehydrogenase
HRP	Horseradish peroxidase
CI	Confidence interval
PT	Pre-treatment
TGE	Tris-glycine electrophoresis
DAB	3,3′-diaminobenzidine
ER	Estrogen receptor
qPCR	Quantitative real time polymerase chain reaction
pCR	Pathological complete response
