# Supplementary material for: DNA Replication Licensing Protein MCM10 Promotes Tumor Progression and Is a Novel Prognostic Biomarker and Potential Therapeutic Target in Breast Cancer
Source: Cancers (Basel). 2018 Aug 22;10(9):282. doi: 10.3390/cancers10090282 (PMC6162382; doi:10.3390/cancers10090282)
Supplement: Supplementary file 1 [file cancers-10-00282-s001.pdf]

Supplementary Materials: DNA Replication Licensing Protein MCM10 Promotes Tumor Progression and Is a Novel Prognostic Biomarker and Potential Therapeutic Target in Breast Cancer

Ravikiran Mahadevappa, Henrique Neves, Shun Ming Yuen[1], Muhammad Jameel, Yuchen Bai, Hiu-Fung Yuen, Shu-Dong Zhang, Youzhi Zhu, Yao Lin, Hang Fai Kwok

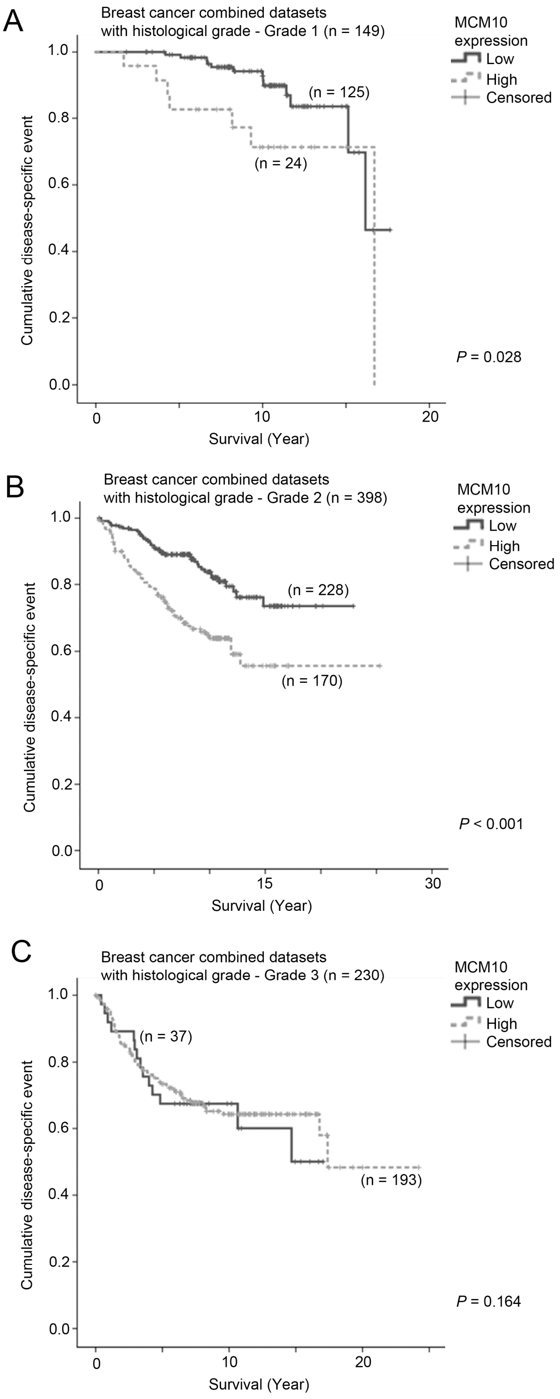

Figure S1. Breast cancer combined datasets with different histological grades.

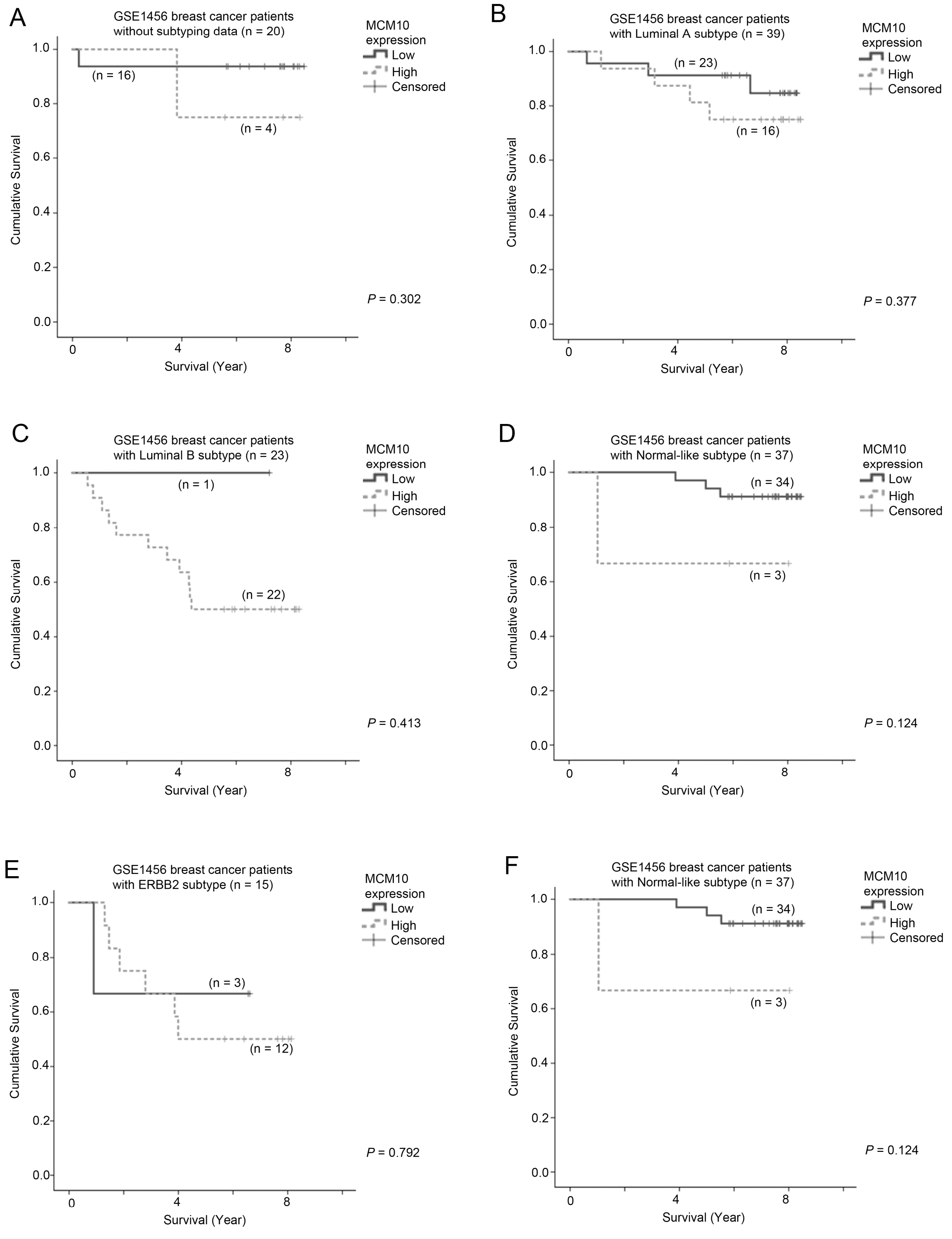

Figure S2. Breast cancer patient different subtypes.

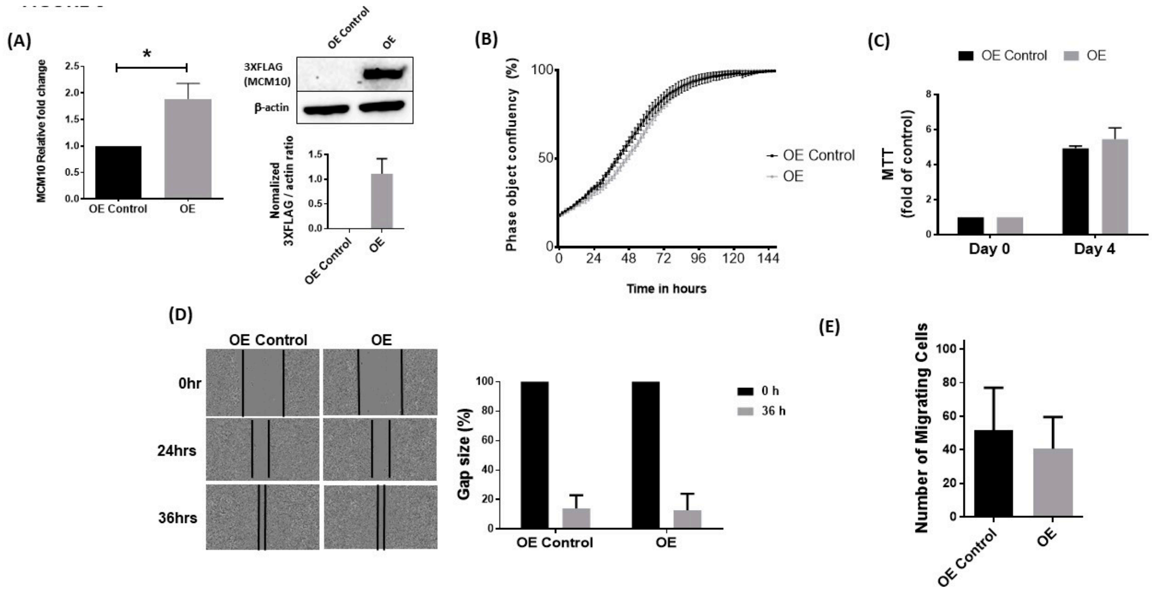

Figure S3. Relative expression of MCM10 in stable Overexpression (OE) in MCF 7 cell lines. (**A**) MCM10 expression was significantly increased in MCM10-OE MCF 7 cell lines assessed by qPCR and western blots ($n = 3$, * $p < 0.05$). (**B**) Cell proliferation in MCM10-OE MCF 7 cell lines showed no difference compared to OE control cells monitored for 7 days in Incucyte ZOOM analysis ($n = 6$) (**C**) MTT proliferation assay using stable cells showed no difference in proliferation rate of MCM10-OE MCF7 cells compared to OE control cells ($n = 3$). (**D**) Wound heal assay performed by using MCM10-OE MCF 7 in Incucyte ZOOM showed no difference. Wound heal quantified by relative Gap size using Incucyte ZOOM software ($n = 6$). (**E**) Transwell cell migration assay showed a similar observation with no difference in number of migrating cells in MCM10-OE MCF7 cells ($n = 3$).

Table S1. Clinicopathological parameters of patient samples used for qPCR analysis of MCM10.

NO.	Age	Location	Category	Tumor size（cm）	Grade	LND NO.	LNM NO.	Cadherin-E	P120	p63	ER	PR	HER2	ki-67	CK5/6	p53
1	48	Right	IDC	2.5 × 2 × 1.2	II	18	8	+			+, 95%	+, 90%	2+	(+, 20%	−	
2	48	Right	LCIS	2.5 × 1.5 × 1.5		15	0	−	cytoplasm, +	myoepithelium, +	+, 90%	+, 60%	2+	+, 10%	myoepithe lium, +	
3	44	Left	IDC		II	20	0				+, 90%	+, 70%	3+	+, 20%	−	
4	38	Right	IDC	3.2 × 2.5	II	16	12	membrane, +	membrane, +		+, 90%	(+, 95%	−	+, 10%	−	
5	41	Right	IDC	The maximum diameter: 2 cm	II	19	1	membrane, +	membrane, +	−	+, 90%	+, 10%	2+	+, 20%	−	
6	36	Left	IDC		II	21	0	cytoplasm, +		−	+, 95%	+, 80%	3+	+, 30%	−	
7	37	Left	IDC		III	16	0	membrane, +	membrane, +		+, 70%	+, 50%	3+	+, 30%		
8	40	Left	IDC		I	29	0	membrane, +	membrane, +	myoepithelium, −	+, 60%	+, 80%	2+	+, 30%		+, <1%
9	59	Left	IDC	4.0 × 3.0 × 3.0	II	23	0	membrane, +	membrane, +	−	+, >95%	+, 20%	3+	+, 30%		+, <1%
10	56	Right	IDC		I	11	0	membrane, +	membrane, +		+, 100%	+, 90%	-	+, 20%		
11	68	Left	IDC		II	16	0	membrane, +	membrane, +		+, 80%	+, 1%	1+	+, 40%		+, 5%
12	60	Right	IDC	3.5 × 3× 2.6	III	16	0	membrane, +	Membrane, +	−	−	−	1+	+, 85%	+	
13	33	Left	IDC		II	22	9	+	+	−	+, 90%	+, 20%	2+	+, 8%	−	
14	43	Left	IDC			27	27	membrane, +	cytoplasm, +	−	+, 10%	+, 10%	3+	+, 40%		+, <1%
15	60	Left	IDC		II	23	1	+	membrane, +	−	+, 95%	+, 80%	3+	+, 20%	−	
16	34	Left	uncertain		II	24	22	membrane, +	membrane, +	+	+, 20%	+, 5%	−	+, 60%	+	

Note: IDC, Invasive Ductal Carcinoma; LCIS, lobular carcinoma in situ; LND, lymph node dissection; LNM, lymph node metastasis; +, positive; −, negative.

© 2018 by the authors. Licensee MDPI, Basel, Switzerland. This article is an open access article distributed under the terms and conditions of the Creative Commons Attribution (CC BY) license (http://creativecommons.org/licenses/by/4.0/).
